# Prevalence of Masked and White-Coat Hypertension in Pre-Hypertensive and Stage 1 Hypertensive patients with the use of TeleMRPA

**DOI:** 10.5935/abc.20190147

**Published:** 2019-11

**Authors:** Weimar Kunz Sebba Barroso, Audes Diógenes Magalhães Feitosa, Eduardo Costa Duarte Barbosa, Roberto Dischinger Miranda, Andréa Araújo Brandão, Priscila Valverde Oliveira Vitorino, Lúcio Paulo de Souza Ribeiro, Marco Mota Gomes

**Affiliations:** 1 Universidade Federal de Goiás - Programa de Pós-Graduação em Ciências da Saúde, Goiânia, Goiás - Brazil; 2 Universidade Federal de Goiás - Liga de Hipertensão Arterial, Goiânia, Goiás - Brazil; 3 Laboratório de Imunopatologia Keizo Asami - Universidade Federal de Pernambuco, Recife, PE - Brazil; 4 Instituto de Cardiologia - Laboratório de Investigação Clínica (LIC), Porto Alegre, RS - Brazil; 5 Escola Paulista de Medicina - Universidade Federal de São Paulo - Cardiogeriatria, São Paulo, SP - Brazil; 6 Universidade do Estado do Rio de Janeiro - Cardiologia, Rio de Janeiro, RJ - Brazil; 7 Escola de Ciências Sociais e da Saúde - Pontifícia Universidade Católica de Goiás - Ciências Sociais, Goiânia, GO - Brazil; 8 Universidade Federal de Pernambuco - Centro de Informática, Recife, PE - Brazil; 9 Centro Universitário CESMAC - Hospital do Coração, Maceió, AL - Brazil

**Keywords:** Hypertension/diagnosis, Masked Hypertension, White Coat Hypertensin, Hypertension Self-Monitoring, Telemedicine

## Abstract

**Background:**

The diagnosis of arterial hypertension based on measurements of blood pressure in the office has low accuracy.

**Objective:**

To evaluate the prevalence of masked hypertension (MH) and white-coat hypertension through home blood pressure monitoring (HBPM) in pre-hypertensive and stage 1 hypertensive patients.

**Method:**

Retrospective study, of which sample consisted of individuals with BP ≥ 120/80 mmHg and < 160/100 mmHg at the medical office without the use of antihypertensive medication and who underwent exams on the HBPM platform by telemedicine (TeleMRPA) between May 2017 and September 2018. The four-day MRPA protocol was used, with 24 measurements, using automated, validated, calibrated equipment with a memory function.

**Results:**

The sample consisted of 1,273 participants, of which 739 (58.1%) were women. The mean age was 52.4 ± 14.9 years, mean body mass index (BMI) 28.4 ± 5.1 kg/m^2^. The casual BP was higher than the HBPM in 7.6 mmHg for systolic blood pressure (SBP) and 5.2 mmHg for diastolic blood pressure (DBP), both with statistical significance (p < 0.001). There were 558 (43.8%) normotensive individuals; 291 (22.9%) with sustained hypertension; 145 (11.4%) with MH and 279 (21.9%) with white-coat hypertension (WCH), with a diagnostic error by casual BP in the total sample in 424 (33.3%) patients. In stage 1 hypertensive individuals, the prevalence of WCH was 48.9%; in prehypertensive patients, the prevalence of MH was 20.6%.

**Conclusion:**

MH and WCH have a high prevalence rate in the adult population; however, in prehypertensive or stage 1 hypertensive patients, the prevalence is higher. Out-of-office BP measurements in these subgroups should be performed whenever possible to prevent misdiagnosis.

## Introduction

The prevalence of arterial hypertension (AH) in the Brazilian adult population is high and varies according to the studied population and the method of assessment (31% to 35.8%).^1^ For this reason, the accurate diagnosis of the several scenarios related to blood pressure (BP) behavior is crucial for adequate stratification of cardiovascular risk, as well as for the definition of the best treatment strategies.^1,2^

In this context, considerations should be given to the possibility of normotension, masked hypertension (MH), white-coat hypertension (WCH), and sustained AH.^3^ MH is defined by the presence of normal BP in the office but high measurements outside of it; the WCH is defined by high values of BP in the office and normal values in the home measurements. It is noteworthy that MH, WCH and AH are highly prevalent and are related to an increase in cardiovascular morbidity and mortality.^4^ Therefore, they should be investigated and diagnosed, and for this purpose, it is essential to use methods capable of monitoring BP outside the doctor's office environment.^5,6^

Ambulatory (ABPM) or home (HBPM) BP monitoring can be used for home BP measurement, with the latter offering the possibility of providing the necessary information for the appropriate diagnosis with greater comfort and better cost-benefit ratio.^7^

Also, in relation to HBPM, the inclusion of the habitual BP measurements into the patient’s routine has shown an increase in compliance with drug treatment. This benefit seems to be even greater when using telemedicine platforms.^8,9^

An adequate identification of MH and WCH is so important that the main guidelines of AH recommend the use of ABPM or HBPM in the diagnostic investigation whenever possible, emphasizing their use at the initial BP alterations.^1,2,3,7^

The present study is the first national study to evaluate the prevalence of MH and white coat hypertension through the HBPM in prehypertensive and stage 1 hypertensive patients.

## Method

This study was submitted and approved by the Human Research Ethics Committee of Hospital das Clínicas da Universidade Federal de Goiás under CAEE number 99691018.7.0000.5078.

This is a retrospective study that evaluated the data of all patients who underwent exams on the TeleMRPA platform (www.telemrpa.com) from May 2017 to September 2018. Of the total, those who underwent the examination for diagnostic purposes and were not using antihypertensive drugs were selected.

Inclusion criteria were age older than 18 years; individuals assessed on the TeleMRPA platform, without the use of antihypertensive drugs and who had, by the casual measure (mean of two measurements) performed at the clinic on the first day of the protocol, SBP and DBP that met the criteria for the diagnosis of prehypertension (PH) - SBP ≥ 120 mmHg and/or DBP ≥ 80 mmHg and SBP < 140 mmHg and DBP < 90 mmHg; or stage 1 AH - SBP ≥ 140 mmHg and/or DBP ≥ 90 mmHg and SBP < 160 mmHg and DBP < 100 mmHg, according to the Brazilian Guideline of Arterial Hypertension (DBHA, *Diretriz Brasileira de Hipertensão Arterial*).^1^

The TeleMRPA platform was developed as a telemedicine monitoring tool, with characteristics that allow the analysis and filtering of the database according to the scientific questions to be investigated. There was a concern to develop and improve the mathematical algorithm aiming to allow the high quality of the analyzable data, either for the interpretation of the exam or for the development of research projects. In this context, the database protects the identification data of the patient and clinics or health units. Prior to the inclusion of data into the platform, the co-investigators were trained regarding the scientific evidence and methodology of the HBPM, as well as for the use of Omron automatic devices for BP measurements.

The protocol used to obtain home measurements follows the recommendation of the Brazilian guidelines for HBPM,^7^ which advises two measures to be carried out on the first day at the office or clinic (these measures are not used for the analysis of the mean home measurements) and six measurements a day on four consecutive days (three in the morning and three in the evening), with a total of 24 measures to calculate the mean. This mean value is considered normal when lower than 135/85 mmHg (Table 1).^1,2,7,8^

**Table 1:** HBPM protocols according to the Brazilian guidelines for HBPM (□□□/□□: blood pressure measurement).^7^

1^st^ day Medical office/clinic	HBPM	2^nd^ day Home	3^rd^ day Home	4^th^ day Home	5^th^ day Home
**Any time** □□□/□□ □□□/□□	**Morning** Before breakfast	□□□/□□	□□□/□□	□□□/□□	□□□/□□
		□□□/□□	□□□/□□	□□□/□□	□□□/□□
		□□□/□□	□□□/□□	□□□/□□	□□□/□□
	**Night** Before dinner or 2 hours later	□□□/□□	□□□/□□	□□□/□□	□□□/□□
		□□□/□□	□□□/□□	□□□/□□	□□□/□□
		□□□/□□	□□□/□□	□□□/□□	□□□/□□

HBPM: home blood pressure monitoring.

It is recommended, on the first day, when the patient receives the device at the health unit, that the patient be taught the correct handling of the BP measuring device, as well as the technique for adequate and reliable measurement. This recommendation follows the DBHA guidelines.^1^ Subsequently, the patient (or caregiver/companion) is instructed to measure the BP twice a day, following the abovementioned protocol.

Patient data, as well as BP values, were included into the TeleMRPA platform and analyzed for the following variables:

Socio-demographic data: gender, age, sample distribution and BP behavior by geographic regions;

Anthropometric data: BMI using the Quetelet formula (BMI = weight in kg/height in meters^2^).

Blood pressure: mean BP at the clinic (first day), mean of home BP measures (second to fifth days) and mean BP in morning and evening, mean pulse pressure and BP variability.

### Database and statistical analysis

The database was created using the Excel® (Microsoft) software with data imported from the TeleMRPA platform; the numerical codes were typed by three researchers with subsequent cross-checking to identify and correct typing errors.

Continuous variables were presented as mean and standard deviation and categorical variables as absolute and relative frequencies. The Kolmogorov-Smirnov test was used to verify the distribution of the continuous variables. The paired *t*-test was used to compare BP measurements between the casual measurements and HBPM. To compare the frequencies of masked AH between stage 1 and stage 2 prehypertensive patients, the chi-square test was used, which was also used to compare the diagnoses of prehypertension and stage 1 hypertension between the casual BP measure and the HBPM. The significance level was set at p < 0.05. The Stata® software, version 14.0 was used for the analysis.

## Results

**Figure 1:**
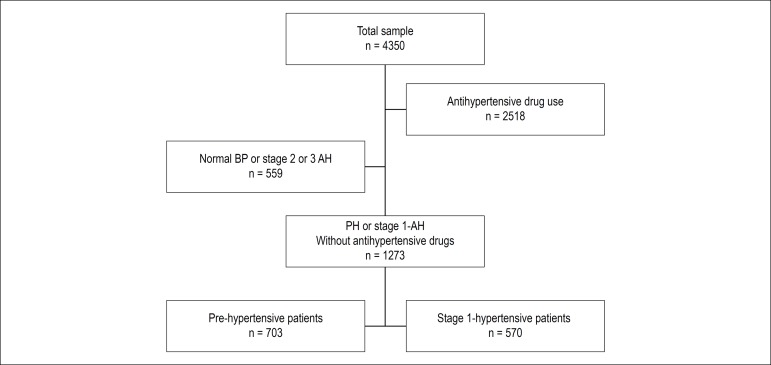
Flowchart for the selection of individuals for the analysis. PH: pre-hypertension, AH: arterial hypertension.

The initial sample consisted of 4,350 individuals who underwent HBPM from May 2017 to September 2018 in nine Brazilian states. Of these, 1,273 participants with a clinical diagnosis of prehypertension or stage 1 AH and without use of medications (Figure 1) were selected, of which 853 (67.0%) were from the Northeast region, 43 (3.4%) from the North region, 10 (0.8%) from the Midwest region, 307 (24.1%) from the Southeast region and 60 (4.7%) from the South region. Mean age was 52.4 ± 14.9 years and the mean BMI was 28.4 ± 5.1 kg/m^2^. As for gender, 739 (58.1%) were women.

The mean values of casual BP were 133.2 ± 11 mmHg and 84.1 ± 8 mmHg, and for HBPM, the mean values were 125.5 ± 11.7 mmHg and 78.9 ± 8 mmHg for SBP and DBP, respectively. The mean number of valid measures was 22.96.

**Figure 2:**
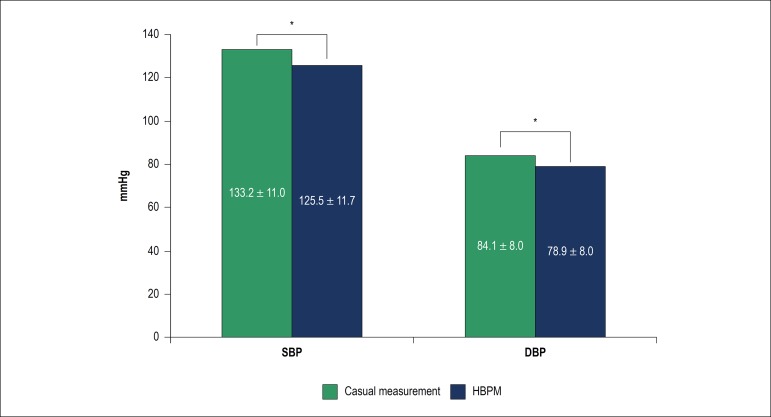
Comparison of systolic and diastolic blood pressure measurements between casual methods and the home blood pressure measurement, n = 1273. Paired t-test *(p < 0.001). SBP: systolic blood pressure; DBP: diastolic blood pressure; HBPM: home blood pressure monitoring.

When comparing the means of the casual BP with the HBPM, higher values were found for casual BP in 7.6 mmHg for SBP and 5.2 mmHg for DBP, both with statistical significance (p < 0.001) (Figure 2).

Considering the casual BP measurement, 703 (55.2%) participants were classified as prehypertensive and 570 (44.8%) as stage 1 hypertensive. When we considered the measures of the HBPM for the diagnosis, in the total sample, 558 (43.8%) were normotensive; 291 (22.9%) had sustained hypertension; 145 (11.4%) had MH; and 279 (21.9%) had WCH; that is, in this population, 33.3% of the diagnoses by casual measurement were wrong.

When analyzing only the prehypertensive group, there were 145 individuals (20.6%) who actually had masked hypertension, and if we separate those individuals with SBP ≥ 130 mmHg and < 140 mmHg, and/or DBP ≥ 85 mmHg and < 90 mmHg (n = 364), the prevalence of MH increases to 27.8% (Table 2).

**Table 2:** Comparison of the prevalence of masked hypertension in pre-hypertensive patients with different blood pressure levels

Blood pressure behavior	120 ≥ SBP < 130 and 80 ≥ DBP < 85 mmHg (casual measurement) n(%)	130 ≥ SBP < 140 e 85 ≥ DBP < 90 mmHg (casual measurement) n(%)	p-value
Normotension True hypertension	295 (87.0%)	263 (72.2%)	
Masked Hypertension	44 (13.0%)	101 (27.8%)	* < 0.001
Total	339 (48.2%)	364 (51.8%)	

* Chi-square. SBP: systolic blood pressure; DBP: diastolic blood pressure.

**Figure 3:**
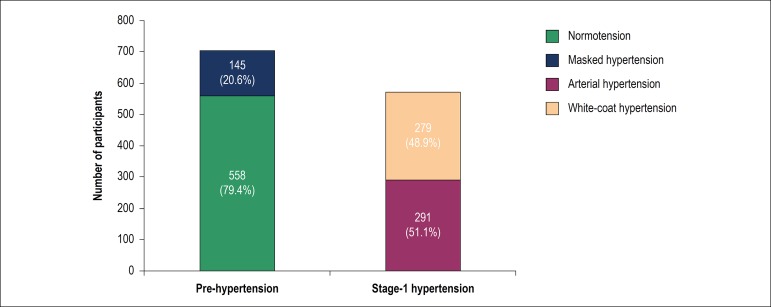
Participants classified as prehypertensive and stage-1 hypertensive patients considering the casual measurement and reclassified according to the HBPM, n = 1,273.

In the stage 1 hypertensive group, 279 individuals (48.9%) with WCH were identified (Figure 3).

## Discussion

The present study confirmed that the BP measurements obtained through HBPM is very useful in the diagnosis of AH phenotypes and allowed the reclassification of 33.3% of the assessed individuals, thus adding important information to the approach and follow-up of these individuals. We emphasize the significant number of individuals included in the study, from nine states of the five Brazilian geographic regions.

AH is a highly prevalent disease in the adult population; in a worldwide survey of 1,128,635 individuals, the prevalence was 34.9%, most of them with stage 1 hypertension. It is also known that the prevalence of PH is at the same level, reinforcing the need for a correct diagnosis, so that the most appropriate conduct can be implemented.^10,11^ It is also well established that BP monitoring methods outside the office, when compared to the casual measure, have a higher diagnostic accuracy and show a better prediction of cardiovascular risk.^12,13^

In the studied sample, when we compared the means of casual BP with HBPM, we found significantly lower means in HBPM, with statistically significant differences (p < 0.001) for both SBP and DBP.

Based on this scientific evidence, to avoid diagnostic error, the most recent AH guidelines have strongly recommended the use of ABPM or HBPM for diagnostic evaluation, especially in individuals with initial BP alterations.^1,2,7^

When only those individuals with diagnostic criteria for PH or stage 1 AH were assessed, in which the chance of casual measures that induce misdiagnosis is higher, we found a prevalence of true normotension and hypertension of only 66.7%, that is, in 33,3% of the cases, the diagnosis would have been wrong, if we considered only the casual measure. These data coincide with other publications that found similar error rates.^14-16^

The risk of WCH in stage 1 hypertensive patients is even more important, or MH in prehypertensive patients because, contrary to what was believed, both WCH and MH are associated with higher cardiovascular mortality and, in the specific case of MH, this mortality is even higher than that of patients with sustained hypertension.^4,17^ It is worth mentioning that this prevalence is high in adults, but it is also significant in adolescents.^6^

In the sample of the present study, among the prehypertensive patients (n = 703), we found a prevalence of MH of 20.6%; this value is even higher when we analyze the highest BP strata in this group. In PH patients with SBP ≥130 mmHg and/or DBP ≥ 85 mmHg, this prevalence is 27.8%, significantly higher (p < 0.001) than PH patients with BP levels lower than 130/85 mmHg. Our findings are in agreement with the trend seen at the last guidelines and statements,^1,2,5,18-21^ which dedicate special attention to prehypertensive individuals, since some of these individuals will show a significant increase in cardiovascular outcomes. It is also justifiable recommending the American and European guidelines regarding the performance of ABPM or HBPM whenever possible when casual BP is ≥ 130/85 mmHg.^2,19^

Finally, when we evaluated the individuals diagnosed as stage 1 hypertension (n = 570), we found a prevalence of WCH of 48.9%, which could result in misdiagnosis and use of inadequate therapeutic strategies in almost half of these individuals.

Most of this sample consisted of individuals from the Northeast region of the country (67.7%); this may be considered a limitation; however, as the analysis refers to AH phenotypes, it seems to us that the geographic region distribution would have little or no effect on the results.

The Telemedicine MRPA (TeleMRPA) platform is an extremely useful, low-cost, easy-to-perform and wide-ranging modality, allowing a systematic evaluation of home-based BP measures, in accordance with the DBHA recommendations. In addition to the individual evaluation in clinical practice, the accumulation of information on this platform represents an important database for analyses on the diagnosis and treatment of AH in several regions of Brazil, both in public and private institutions.

## Conclusion

The casual BP measurement is a method used for AH screening, but it is necessary to take into account that home measurements are more accurate for the diagnosis.

We found high prevalence rates of MH and white-coat hypertension in individuals diagnosed with prehypertension or stage-1 hypertension.

The TeleMRPA platform allowed the reclassification of 33.3% of the assessed individuals.
